# Long-Term Immune Alterations After Thymectomy in Early Childhood: A Case Series

**DOI:** 10.7759/cureus.95517

**Published:** 2025-10-27

**Authors:** Vasiliki M Kymioni, Panagiota Panagiotou, Mariana Tzanoudaki, Vana Spoulou

**Affiliations:** 1 Paediatrics, Agia Sofia Children's Hospital, Athens, GRC; 2 Immunology, Agia Sofia Children's Hospital, Athens, GRC; 3 Paediatric Infectious Diseases, National and Kapodistrian University of Athens School of Medicine, Athens, GRC

**Keywords:** children, digeorge syndrome, immunophenotype alterations, pediatric immunology, thymectomy

## Abstract

The thymus plays a central role in T-lymphocyte maturation and in maintaining immune defense against infections and autoimmunity. We describe three boys (mean age 7.3 years) who underwent total thymectomy during surgical correction of congenital heart disease, two in infancy and one in the neonatal period. Patient A presented (14 years old) with recurrent cervical lymphadenopathy, generalized Epstein-Barr virus (EBV) infection, and hepatic fibrosis. Patient B (two-year-old) developed splenomegaly and had a history of frequent lower respiratory tract infections and pseudohypoaldosteronism type II. Patient C (seven-year-old) presented with generalized lymphadenopathy, hepatosplenomegaly, neutropenia, and thrombocytopenia; his medical history included skeletal anomalies and immune cytopenia with response to intravenous immunoglobulin, but no severe infections. All three patients displayed significantly low naïve CD4 counts, confirming the existence of long-term immune changes following thymectomy. They shared the same immunophenotype, characterized by significantly low naive CD4 cells, which is considered a marker of long-term immune dysregulation following thymectomy. However, their clinical presentation varied, encompassing a spectrum of manifestations ranging from recurrent viral infections to severe immune cytopenias. The immunophenotypic similarities to 22q11.2 deletion syndrome underscore the need to include surgical thymectomy in the differential diagnosis of T-cell dysregulation. Notably, the most severe immune abnormalities were seen after thymectomy during the neonatal period, emphasizing the crucial influence of surgical timing. Long-term immunological monitoring of these children is warranted to anticipate and manage infections or immune-mediated complications.

## Introduction

The thymus plays a key role in T-cell differentiation, T-cell repertoire selection, and immune system development from fetal life to early childhood [1,2]. It is a common practice to perform thymectomy during cardiac surgeries to achieve a better surgical window. Following thymectomy, there have been reports of long-term immunological effects [3-6]. A later-life propensity to recurrent infections and immune dysregulation has been associated with decreased naïve T-cell regeneration and naïve/memory ratios, as well as worse immunological control [4,5]. These changes are thought to result from reduced thymic emigrant production and compensatory homeostatic proliferation, which maintain T-cell counts but may narrow T-cell repertoire diversity [6-9].

As a significant number of children who underwent thymectomy in infancy are reaching adolescence and adulthood, there is growing interest in understanding their long-term clinical and immunological profiles [4]. However, current evidence remains limited, with most studies focusing on immunophenotypic changes rather than comprehensive long-term clinical outcomes. Data on infection susceptibility, autoimmune manifestations, and correlations between immune profiles and clinical findings are particularly scarce. Further research and detailed case reports are therefore essential to better characterize post-thymectomy immune dysregulation and its clinical implications.

In this report, we describe three cases of boys who experienced severe, persistent, or recurrent infections after undergoing thymectomy early in life. We examine their immunological characteristics, explain the variation in their clinical outcomes, and compare them with patients who have 22q11.2 deletion syndrome (DiGeorge syndrome).

## Case presentation

Patient A

A 14-year-old male with congenital heart disease (CHD) - transposition of the great arteries (TGA) - underwent multiple corrective surgeries, including total thymectomy at 18 months of age. He was fully immunized and had a normal karyotype (46, XY) with a negative 22q11.2 FISH result.

**Figure 1:**
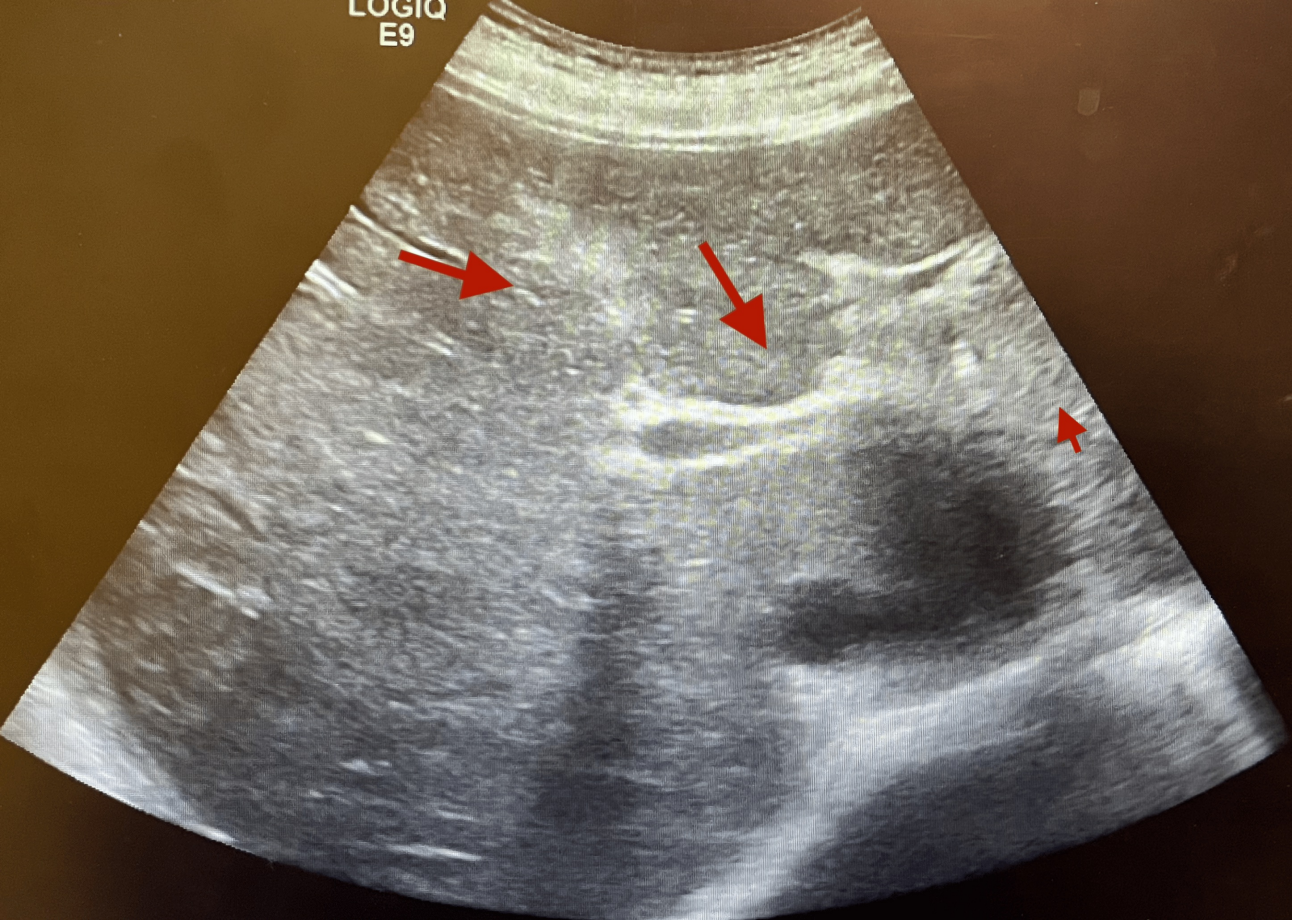
Areas of hepatic fibrosis are marked by red arrows

He presented with intermittent cervical lymphadenopathy, eyelid edema, and frequent respiratory infections. More precisely, he underwent recurrent sinus infections (one to two episodes annually) and recurrent pneumonia, with one episode per year during the past three years, requiring antibiotic therapy. Clinical evaluation revealed multiple enlarged cervical lymph nodes. The work-up ruled out autoimmune disease and malignancy, while abdominal ultrasound and laboratory results showed hepatic fibrosis and widespread Epstein-Barr virus (EBV) infection (Figure 1).

Immunological evaluation with flow cytometry showed normal CD4 T, B, and NK cell counts, but a markedly reversed naïve/memory CD4 ratio (9/91), low absolute CD8 T-cells (108/μL), and an increased CD4/CD8 ratio (4.9).

Patient B 

A male patient with TGA underwent corrective surgery, including thymectomy, at 16 months of age. He was also diagnosed with CUL3-related pseudohypoaldosteronism type II.

He presented at two years of age with splenomegaly and a history of frequent lower respiratory infections. His medical history was notable for one to two pneumonia episodes per year, with at least one hospitalization annually for intravenous antibiotics, consistent with recurrent infections suggestive of immune dysfunction.

Immunological evaluation, including lymphocyte subset analysis, revealed significant T lymphopenia (692/μL) that partially improved with age. The CD4/CD8 ratio was conserved, while the NK cell count was at the lower limit of normal (59/μL). The naïve/memory CD4 ratio was reversed (33/67) but improved over time. B-cell counts and T-cell activation markers were within normal limits.

Patient C 

A male infant diagnosed with complex CHD (interrupted aortic arch type I, right aortic arch, incomplete vascular ring, large ventricular septal defect) underwent cardiac surgery with thymectomy at 20 days of age. He also had skeletal anomalies, including radial hypoplasia, scoliosis, and coarse vertebrae.

At seven years of age, he developed generalized lymphadenopathy, hepatosplenomegaly, neutropenia, and thrombocytopenia, the latter responding to intravenous immunoglobulin. No serious infections were reported. The work-up excluded myelodysplasia, autoimmune disease, and an underlying genetic syndrome.

Immunologic evaluation with flow cytometry revealed normal total T, B, and NK cell numbers, with NK cell counts at the upper limit of normal (902/μL). The CD4/CD8 ratio and TCRγδ expression were normal. However, the naïve/memory CD4 ratio was profoundly reversed (10/90), with reduced switched memory B-cells (2.1%) and expansion of IgD⁺CD27⁺ memory B-cells without isotype switching (20.2%).

Tables 1-2 provide a concise summary of the clinical and immunophenotypical features of the patients. 

**Table 1:** Cases summary EBV, Epstein-Barr virus; IVIG, intravenous immunoglobulin; PDA, patent ductus arteriosus; TGA, transposition of the great arteries; VSD, ventricular septal defect

Patient	Age at thymectomy	Reason for surgery	Clinical course
A	18 months	Correction of TGA	Recurrent cervical lymphadenopathy, EBV infection with hepatic fibrosis, frequent respiratory infections
B	16 months	Correction of TGA	Splenomegaly at two years, frequent lower respiratory infections, pseudohypoaldosteronism type II
C	20 days	Complex congenital heart disease (interrupted aortic arch, VSD, PDA, vascular ring)	Generalized lymphadenopathy, hepatosplenomegaly, cytopenia (responding to IVIG), skeletal anomalies

**Table 2:** Immunophenotypic comparison of patients Additional immunophenotypic parameters (CD4⁺CD45RA⁺ absolute counts and B-cell memory subsets) were included to provide a more detailed characterization of T- and B-cell compartment alterations.

Parameter	Patient A	Patient B	Patient C
Total T-cells	Normal for age	Significant lymphopenia (692/µL, improved with age)	Normal for age
CD4 T-cells	Normal	Normal	Normal
CD8 T-cells	Low	Normal	Normal
CD4/CD8 ratio	Increased	Preserved	Normal
Naïve/memory CD4 ratio	Inverted (9/91)	Inverted (33/67, improving)	Inverted (10/90)
CD4+CD45+RA+ ratio on total CD4+	8,6%	10%	21%
CD4+CD45+RA+/μL	45/μL	91/μL	42/μL
CD19+ B-cells	Normal	Normal	Normal
CD27+IgD-(switched memory cells) ratio on B-cells	8.3%	4.1%	7.6%
CD27+IgD+ratio on B-cells (non-switched memory cells)	2%	17.3%	7.6%
NK cells	Normal	Lower limit of normal (59/µL)	High (902/µL)
Other subsets	-	-	Expansion of IgD+CD27+ memory B-cells

## Discussion

Our case series highlights that early thymectomy produces a lasting impact on immune homeostasis. Although our patients displayed comparable immunophenotypic patterns, they experienced varied clinical courses, emphasizing the diversity of post-thymectomy outcomes. These findings pave the way for comparing our results with those of previous studies and investigating the factors that may influence immune recovery.

Our findings align with the immune alterations observed in infants. All three patients in our series demonstrated markedly low CD4 naïve cell counts. Patient B exhibited persistent lymphopenia, while Patients A and C maintained near-normal T-cell counts. This variability reflects the results of earlier reports, which emphasize that long-term immune reconstitution after thymectomy is heterogeneous [4,10-12].

The impact of T-lymphopenia has additional consequences. Several studies have documented disruptions in B-cell maturation, decreased germinal center activity, and altered antibody responses [4,13]. The consistently low absolute CD4⁺CD45RA⁺ counts across patients, despite near-normal total CD4 counts, emphasize the significant and enduring reduction in thymic output. In our cohort, total B-cell counts and immunoglobulin levels, when measured, were within the expected range for age. The reduction in switched memory B-cells and the increase in unswitched memory B-cells in some patients indicate impaired germinal center responses. This observation has been reported in other groups of thymectomized patients. However, Patient C exhibited reduced switched memory B-cells, along with an increase in unswitched memory subsets. NK-cell counts remained stable or even increased, consistent with previously described compensatory changes (Table 2) [9,14]. 

Clinically, thymectomy has been associated with increased susceptibility to infections, poor vaccine responses, and immune dysregulation [15-18]. Our patients demonstrate this variability: Patient A experienced recurrent infections and EBV-related hepatitis and fibrosis; Patient B developed frequent respiratory infections and splenomegaly; while Patient C presented with immune cytopenia but did not suffer severe infections. These contrasting outcomes highlight that similar immunophenotypes may translate into very different clinical trajectories.

Autoimmune complications have also been reported in thymectomized patients, often attributed to reduced regulatory T-cell activity, and they frequently appear later in life [4,19]. None of our patients developed frank autoimmune disease, although the cytopenia in Patient C may represent early immune dysregulation. Comparable cases of cytopenia and autoantibody production after thymectomy have been documented [5,14].

Taken together, our patients’ findings are consistent with the growing body of evidence describing long-term immune consequences after early thymectomy. Beyond the well-documented alterations in T-cell numbers and subsets, several studies have reported important clinical outcomes. Children and young adults who have undergone thymectomy appear more prone to respiratory infections, likely reflecting reduced thymic output and a restricted T-cell repertoire [4,5,12,13]. In some cases, immune dysregulation or autoimmune manifestations may also develop, possibly due to impaired central tolerance and altered regulatory T-cell function [3,4,6,7,10,13]. Although a definite link with malignancy or lymphoproliferative disease has not been firmly established, persistent immune imbalance could theoretically contribute to such complications, highlighting the importance of long-term clinical monitoring [4,5,7,12,13].

The immunological features observed in thymectomized patients show notable overlap with those seen in genetic thymic insufficiency, particularly in 22q11.2 deletion syndrome. Both conditions are characterized by reduced naïve T-cells, an inverted naïve-to-memory cell ratio, and expansion of memory subsets [1,2,20]. Unlike patients with 22q11.2 deletion, however, thymectomized children generally lack syndromic features and often maintain normal immunoglobulin levels [2,20]. None of our patients had a 22q11.2 deletion, yet their immunological findings closely resembled those reported in affected individuals (Table 3).

**Table 3:** Comparison of 22q11.2 Deletion Syndrome with our case series [1,2,20]

Feature	22q11.2 Deletion Syndrome	This case series
Genetic basis	22q11.2 microdeletion (TBX1, others)	No genetic abnormality detected
Thymus	Hypoplasia/aplasia	Surgical removal (complete)
Congenital anomalies	Cardiac defects, craniofacial anomalies, hypocalcemia, skeletal anomalies	Only cardiac defects (Patient C also skeletal anomalies, but negative genetics)
T-cells	Reduced total T-cells; low naïve T-cells; inverted naïve/memory ratio	All had inverted naïve/memory ratio; variable total T-cell counts (Patient B lymphopenia, A and C near-normal)
B-cells	Generally normal	Normal counts; Patient C had reduced switched memory B-cells
Immunoglobulins	Usually normal, though variable	Normal for age
NK cells	Preserved	Normal or increased
Clinical manifestations	Recurrent infections, autoimmune disease, variable severity	Recurrent infections (A, B); cytopenias (C); no autoimmune disease detected

Finally, the age at thymectomy is a critical factor in determining the clinical course of children. Surgery performed in the first weeks of life is associated with more severe and persistent lymphopenia [10,11,12,17]. This was evident in our cohort: Patient C, thymectomized at 20 days of life, showed more pronounced immune abnormalities than patients operated later in infancy. Long-term studies confirm that these alterations can persist into adulthood [20], underscoring the importance of careful, ongoing follow-up for children undergoing early thymectomy.

This study has some limitations. The relatively young age of the patients means that their immune and clinical profiles are still evolving, and the full extent of long-term effects may not yet be apparent. In addition, the small number of cases limits how broadly these findings can be applied and prevents firm conclusions about causality.

## Conclusions

Thymectomy in early life leaves a clear and lasting mark on immune development, most notably by skewing the T-cell compartment toward memory phenotypes at the expense of naïve cells. Our three patients illustrate that this pattern is consistent; however, its clinical impact is not uniform, as some children remain relatively well, while others develop infections, viral complications, or immune cytopenia. The overlap with 22q11.2 deletion syndrome underscores the importance of considering thymectomy in the differential diagnosis of T-cell dysregulation, particularly when syndromic features are absent. The timing of thymectomy appears to be a critical determinant, with neonatal surgery linked to more pronounced and enduring immune changes. These observations underscore the need for careful and ongoing immunological follow-up in children who undergo thymectomy during the earliest stages of life.
